# Ganglionic Plexus Ablation During Pulmonary Vein Isolation - Predisposing to Ventricular Arrhythmias?

**Published:** 2010-02-01

**Authors:** Faizel Osman, Suman Kundu, Jiun Tuan, Mohamed Jeilan, Peter J Stafford, G Andre Ng

**Affiliations:** 1 Department of Cardiology, University Hospital Coventry, Clifford Bridge Rd, Coventry CV2 2DX, UK; 2 Department of Cardiology, Glenfield Hospital, University Hospitals of Leicester, Leicester LE3 9QP, UK

**Keywords:** Catheter ablation, Atrial fibrillation, Vagal denervation, Ventricular fibrillation

## Abstract

Catheter ablation is increasingly used to treat patients with atrial fibrillation (AF). Ablation of ganglionic plexi is often performed to reduce vagal innervation and has been shown to confer a better long-term outcome in terms of AF recurrence. We report a case of a patient having AF ablation with a profound vagal response, suggesting ganglionic plexus ablation, who subsequently developed ventricular fibrillation after programmed ventricular stimulation. Reduced vagal modulation is known to predispose to ventricular arrhythmias and vagal denervation following AF ablation may predispose to ventricular arrhythmias and requires further study.

## Introduction

Catheter ablation is increasingly used to treat patients with atrial fibrillation (AF) [1]. During ablation bradycardia and asystole may occur, which have been interpreted as effects from vagal stimulation around the ablation sites. Reports suggest that ablation at these sites with subsequent vagal denervation and abolition of such reflexes confer a better long term procedural result [2]. We report a case where such vagal responses were elicited during catheter ablation in a patient with paroxysmal AF (PAF). Programmed ventricular stimulation conducted post-procedure unexpectedly provoked polymorphic ventricular tachycardia (VT) followed by ventricular fibrillation (VF), which was terminated with external electrical cardioversion.

## Case Report

**Figure 1:**
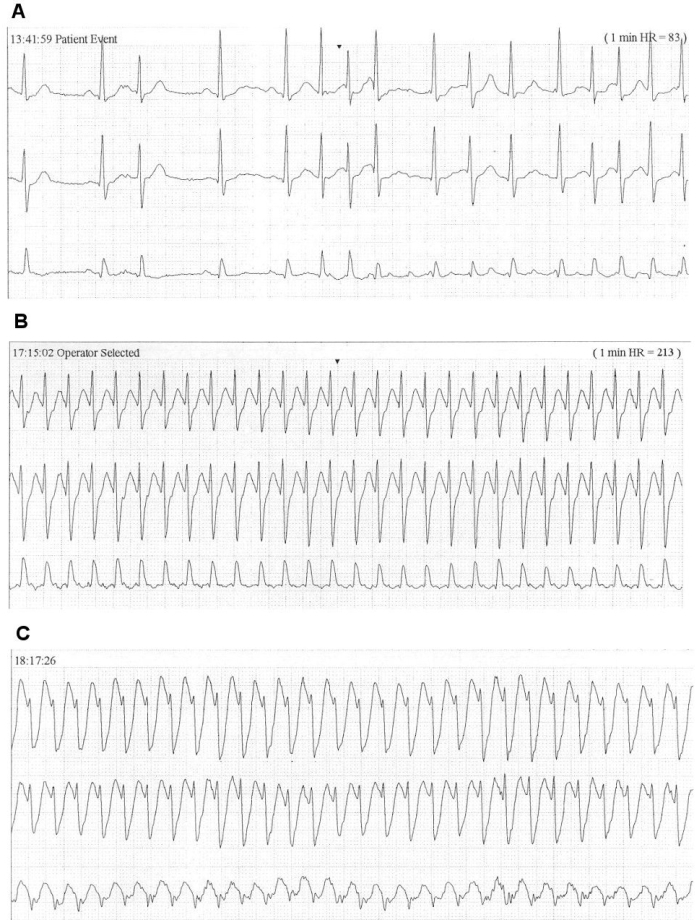
Holter recording showing a) P on T atrial ectopics triggering an episode of atrial fibrillation, b) a regular narrow complex tachycardia with a rate of 210 beats/minute and c) a broad complex tachycardia with a similar rate.

A 55-year old man with no past medical history was found to be in fast AF following a leg injury. He underwent successful external cardioversion following a normal trans-esophageal echocardiogram; the latter revealed no evidence of intra-cardiac thrombus, preserved biventricular systolic function and no significant valvular lesions. His CHADS2 score was 0 and he was commenced onto warfarin therapy following electrical cardioversion. The AF recurred 36 hours later and he was commenced on oral flecainide which reverted him back to sinus rhythm but his palpitation continued. Subsequent Holter monitoring revealed episodes of paroxysmal AF triggered by atrial premature beats (APBs), a narrow complex tachycardia and a regular broad complex tachycardia at a similar rate thought to represent a supraventricular tachycardia with aberrant conduction (Figure 1). As he remained symptomatic despite maximal dose of flecainide he was changed to sotalol and his symptoms improved. Repeat Holter monitoring showed APBs and occasional ventricular premature beats (VPBs). Cardiac catheterization revealed good left ventricular systolic function with normal coronary arteries.

**Figure 2:**
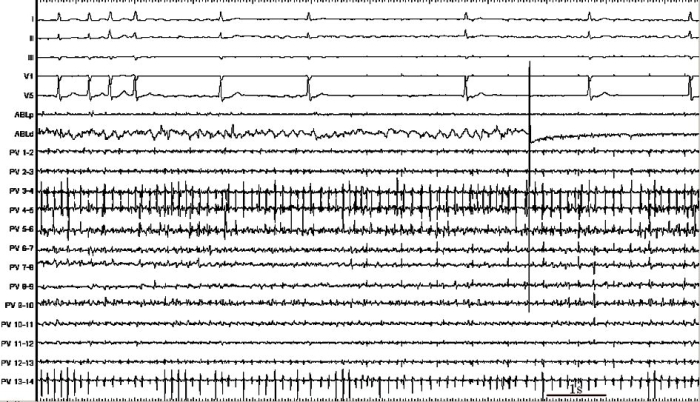
Profound bradycardia was induced during ablation of the pulmonary veins. Simultaneous recordings in ECG leads I, II, III, V1 and V5 and intracardiac signals from the proximal (ABLp) and distal (ABLd) bipoles of the ablation catheter and the 7 pairs of bipolar electrograms from the circular pulmonary vein catheter (PV 1-14).

**Figure 3:**
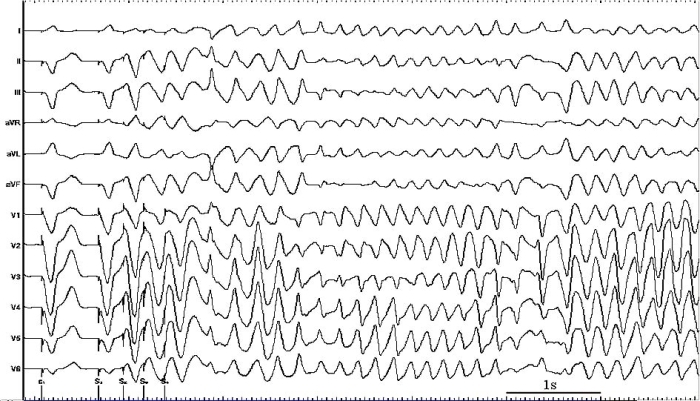
Twelve-lead ECG recording during the ventricular stimulation study with 3 extrasystoles (immediately after catheter ablation) inducing polymorphic ventricular tachycardia which degenerated into ventricular fibrillation.

Unfortunately, he remained symptomatic with PAF and was unwilling to commence amiodarone given its long-term side effects and opted for percutaneous catheter ablation therapy. Baseline electrophysiology study revealed concentric decremental retrograde and antegrade conduction; dual AV node physiology was not demonstrated and tachycardia was not induced despite use of intravenous isoprenaline infusion, repeat curves and burst pacing. Given his documented PAF, pulmonary vein isolation (PVI) was performed using the Ensite NavX™ electro-anatomic mapping system (St Jude Medical, Minnesota, USA). Ablation was carried out at the ostia of all four pulmonary veins. During the ablation procedure the patient went into AF; ablation was carried out near areas of complex fractionated atrial electrograms around the left superior and inferior pulmonary veins which produced profound bradycardia (Figure 2). All four pulmonary veins were successfully isolated and sinus rhythm resumed spontaneously. Due to the previously documented broad complex tachycardia on Holter monitoring, programmed ventricular stimulation was performed which, unexpectedly, produced polymorphic VT degenerating into VF (Figure 3). This occurred during 600 ms drive cycle with 3 ventricular extrasystoles (at coupling intervals 260, 220 and 240 ms). An external 200J biphasic shock restored sinus rhythm. The patient was well post-procedure. Subsequent cardiac magnetic resonance imaging was entirely normal. Repeat programmed ventricular stimulation 4 months later failed to induce further arrhythmia. The patient remains symptom free at follow-up, off all anti-arrhythmic medication, with no arrhythmia on Holter monitoring and heart rate variability (HRV) parameters (SDNN, RMSSD and pNN50) were normal.

## Discussion

During catheter ablation for AF, bradycardic responses are often seen, especially at and around the pulmonary veins. These have been regarded as 'vagal reflexes' and abolition of these reflexes during ablation are believed to represent vagal denervation [2]. These patients have been shown to have attenuated heart rate variability consistent with vagal withdrawal and found to be less likely to have recurrent AF up to 3 months after ablation [2]. Localized cardiac autonomic ganglionated plexi may also play a role in AF initiation and maintenance [3] and adjunctive targeted vagal denervation during AF ablation has been proposed.

Cardiac autonomic modulation is known to have a significant influence on initiation of ventricular arrhythmias and sudden cardiac death. We recently reported that vagal stimulation decreases susceptibility of the heart to VF, while sympathetic stimulation increases it [4]. Disturbance of cardiac autonomic function with catheter ablation has been reported with ablation at atrial septal sites and inappropriate sinus tachycardia following ablation has been shown to be due to withdrawal of parasympathetic activity with spontaneous recovery 3 months later [5]. Destruction of cardiac innervation at the atrial level may also damage ventricular innervation downstream [6]. The response to programmed ventricular stimulation in our patient may have been due to the disturbance of cardiac autonomic modulation as a result of ablation of ganglionic plexi. Lack of inducibility of ventricular arrhythmia 4 months later using the same stimulation protocol suggests catheter ablation at the atrial level directly affected cardiac autonomic function and possibly increased the susceptibility of the myocardium to ventricular arrhythmia immediately post-ablation, an effect which resolved 4 months later with resolution of cardiac autonomic function. We performed a VT stimulation study after PVI. Whether we would have induced a ventricular arrhythmia pre-ablation is unknown. We routinely perform a baseline EP study in all patients in sinus rhythm prior to PVI. Whether a VT stimulation study should be performed as well remains to be evaluated.

The strategy of vagal denervation using ganglionic plexus ablation during AF ablation to improve long term success requires further study. Medium and long term follow up to exclude a predilection to ventricular arrhythmias is required to ensure no deleterious long term effects.
